# Hippocampal Availability of the α7 Nicotinic Acetylcholine Receptor in Recent-Onset Psychosis

**DOI:** 10.1001/jamanetworkopen.2024.27163

**Published:** 2024-08-12

**Authors:** Nicole R. Wong, Leah H. Rubin, Courtney K. Harrington, Katelyn R. Jenkins, Laura K. Shinehouse, Mark Yoon, Jessica J. Kilgore, Ana R. Soule, Wojciech G. Lesniak, Steven P. Rowe, Andrew G. Horti, Vidyulata Kamath, Robert F. Dannals, Yong Du, Martin G. Pomper, Jennifer M. Coughlin

**Affiliations:** 1 Department of Psychiatry and Behavioral Sciences, Johns Hopkins University School of Medicine, Baltimore, Maryland; 2 Department of Neurology, Johns Hopkins University School of Medicine, Baltimore, Maryland; 3 Department of Epidemiology, Johns Hopkins University School of Medicine, Baltimore, Maryland; 4 Department of Molecular and Comparative Pathobiology, Johns Hopkins University School of Medicine, Baltimore, Maryland; 5 The Russell H. Morgan Department of Radiology and Radiological Science, Johns Hopkins University School of Medicine, Baltimore, Maryland; 6 Department of Radiology, University of North Carolina, Chapel Hill

## Abstract

**Importance:**

Studies using human postmortem tissue and imaging with positron emission tomography (PET) support a low hippocampal availability of the α7 nicotinic acetylcholine receptor (α7-nAChR) in psychotic conditions, particularly in schizophrenia or schizoaffective disorder (nonaffective psychosis). If validated further, the finding may have implications for clinical diagnosis and treatment.

**Objective:**

To test for lower availability of the α7-nAChR in the hippocampus of individuals with recent-onset psychosis compared with healthy control individuals and its association with lower cognitive performance or higher psychotic symptom burden within recent-onset psychosis.

**Design, Setting, and Participants:**

In this cross-sectional study, healthy individuals without history of psychosis and patients within 10 years of a first onset of psychotic disorder were recruited from the greater Baltimore, Maryland, and Washington, DC, area. Fluorine 18–labeled ASEM ([^18^F] ASEM) PET data were acquired from participants enrolled between March 1, 2014, and July 31, 2023, from an academic research institution. Data acquired between March 1, 2014, and January 31, 2018 (n = 26), were published as a pilot study and were combined with new data acquired between January 1, 2019, and July 31, 2023 (n = 33).

**Main Outcome and Measures:**

Regional [^18^F]ASEM total distribution volume (V_T_) that measures α7-nAChR availability, global cognition composite score, and total scores on the Scale for the Assessment of Positive Symptoms and the Scale for the Assessment of Negative Symptoms.

**Results:**

A total of 59 participants (30 women [51%]; mean [SD] age, 25.5 [5.2] years), including 35 with recent-onset psychosis and 24 healthy controls, completed the study. In age-adjusted analyses, lower hippocampal [^18^F]ASEM V_T_ was found in individuals with recent-onset psychosis (mean [SE], 17.87 [0.60]) compared with healthy controls (mean [SE], 19.82 [0.73]) (*P* = .04). In addition, [^18^F]ASEM V_T_ was lower in individuals with nonaffective psychosis (mean [SE], 16.30 [0.83]) compared with healthy controls (*P* = .006) or those with affective psychosis (mean [SE], 19.34 [0.80]) (*P* = .03). Across recent-onset psychosis and after controlling for age, lower hippocampal [^18^F]ASEM V_T_ was associated with more positive (*r* = −0.44; *P* = .009) but not negative symptoms, and higher hippocampal V_T_ was associated with better global cognition composite score (*r* = 0.38; *P* = .03).

**Conclusions and Relevance:**

In this cross-sectional study of individuals with recent-onset psychosis compared with healthy controls, a lower hippocampal α7-nAChR availability was found in recent-onset psychosis, and its availability was lower in those with nonaffective vs affective psychosis. Further study of the association between low availability of the α7-nAChR and recent-onset psychosis is warranted toward informing diagnostic or therapeutic strategies related to these findings.

## Key Points

**Question:**

Is availability of the α7 nicotinic acetylcholine receptor (α7-nAChR) low in the hippocampus of individuals with recent-onset psychosis?

**Findings:**

In this cross-sectional study of 59 participants (35 with recent-onset psychosis and 24 healthy controls), fluorine 18–labeled ASEM positron emission tomography revealed lower hippocampal availability of α7-nAChR in individuals with recent-onset psychosis compared with healthy individuals, with lowest availability in those with nonaffective vs affective psychosis. Within patients, low availability of the α7-nAChR was associated with positive symptoms and lower cognitive performance.

**Meaning:**

These findings suggest a low availability of the α7-nAChR in recent-onset psychosis, which may be most robust in nonaffective psychosis and linked to clinical signs, and suggest a need for further research toward informing diagnostic or therapeutic strategies.

## Teaser

This cross-sectional study assesses hippocampal availability of the α7 nicotinic acetylcholine receptor (α7-nAChR) among participants with recent-onset affective and nonaffective psychosis compared with healthy individuals.

## Introduction

Converging lines of evidence support low expression and/or activity of the α7 nicotinic acetylcholine receptor (α7-nAChR) in schizophrenia. Genetic variants within the promoter of the α7-nAChR gene (*CHRNA7* [118511]) have shown linkage to deficient sensory gating seen in schizophrenia,^1^ and deletions in *CHRNA7* are associated with a high risk for the disorder.^2,3^ A low expression of the α7-nAChR across the brains of individuals with schizophrenia may result from alterations in *CHRNA7*.^4^ Postmortem studies showed low α7-nAChR expression in hippocampus, cingulate cortex, frontal lobe, and the thalamic reticular nucleus of cases of schizophrenia.^5,6,7,8,9^ Mechanistic models of schizophrenia posit that deficient activity of the α7-nAChR on γ-aminobutyric acid (GABA)–ergic interneurons may disinhibit excitatory inputs to glutamatergic pyramidal cells to drive signs and symptoms of the condition.^10^ However, further study of α7-nAChR in the brains of individuals with schizophrenia is needed, since postmortem findings may be limited by confounding factors (disease chronicity, lifetime medications, or nicotine exposure) or undocumented clinical metrics (positive or negative symptom severity or cognitive performance) at the time of death.

Studying the availability of the α7-nAChR in the human brain in vivo, Horti et al^11,12^ developed fluorine 18–labeled ASEM ([^18^F]ASEM) for use with positron emission tomography (PET). [^18^F]ASEM has high binding affinity (inhibitory constant, 0.37 nM) for the α7-nAChR^11^ and reversible pharmacokinetics in the human brain.^13,14^ [^18^F]ASEM PET revealed a pattern of rising α7-nAChR availability over healthy aging^14^ and higher availability of α7-nAChR in individuals with mild cognitive impairment relative to age-matched, cognitively intact individuals.^15^ In an in vivo pilot study using [^18^F]ASEM PET, individuals with recent-onset psychosis had lower hippocampal availability of the α7-nAChR compared with healthy individuals.^16^ Within recent-onset psychosis, lower hippocampal α7-nAChR availability was found in individuals with schizophrenia or schizoaffective disorder (collectively grouped nonaffective psychosis [NP]) compared to bipolar I disorder (affective psychosis [AP]).^16^ Those data suggest there may be a link between low α7-nAChR availability in the hippocampus and enduring, psychotic symptoms that occur independently of affective episodes. Furthermore, within recent-onset of psychosis (AP plus NP), lower hippocampal α7-nAChR availability was associated with lower cognitive performance, suggesting the relevance of this imaging marker to dimensional psychopathology across psychotic conditions.

Herein we report findings from use of [^18^F]ASEM PET in an expanded, transdiagnostic study of recent-onset psychosis. We hypothesized a lower hippocampal availability of the α7-nAChR in recent-onset psychosis compared with healthy control individuals, with lowest hippocampal availability in NP. Within recent-onset psychosis, we hypothesized that low hippocampal availability of the α7-nAChR would be associated with lower cognitive performance and higher psychotic symptom burden.

## Methods

### Human Participants

This study protocol was approved by the Johns Hopkins University Institutional Review Board. Results were reported in accordance with the Strengthening the Reporting of Observational Studies in Epidemiology (STROBE) guideline. Each participant provided written informed consent. Participants were enrolled between March 1, 2014, and July 31, 2023. Data from the first 26 individuals (acquired between March 1, 2014, and January 31, 2018) were published as a pilot study^16^ and were combined with data from 33 new individuals acquired between January 1, 2019, and July 31, 2023. Participants were primarily recruited from the greater Baltimore, Maryland, and Washington, DC, area. Advertisements sent through the electronic medical record were used for recruitment.

Each individual completed the diagnostic Structured Clinical Interview for *DSM-IV*^17^ with a trained study team clinician (A.R.S. or J.M.C.). Eligibility criteria were broadened after the published pilot phase^16^ to lengthen the time since onset in the definition of recent-onset psychosis, allow treatment with more than lithium or antipsychotic monotherapy (polypharmacy allowed), and permit inclusion of cannabis or limited, recent nicotine use (described later). In the original pilot study,^16^ individuals were included if they were found on the Structured Clinical Interview for *DSM-IV* to be either a healthy individual without a history of psychosis or within 5 years of onset (recent onset) of schizophrenia or schizoaffective disorder (NP) or bipolar I disorder (AP). In the original pilot, exclusion criteria consisted of (1) any psychotropic medication use other than monotherapy with either lithium or an antipsychotic medication that was allowed in individuals with psychosis; (2) contraindication to magnetic resonance imaging (MRI) or PET; (3) lack of English proficiency (due to possible influence on neuropsychological performance); (4) active use of nicotine; (5) current misuse of alcohol or recreational substances, including cannabis (assessed by self-report and urine toxicology); and (6) any clinically significant abnormality on laboratory blood test results, electrocardiography, or structural MRI.

After the pilot study, the definition of recent onset was broadened to include individuals within 10 years of onset of the psychotic condition. With expansion to permit cannabis or limited, recent nicotine use, the relevant exclusion criteria (No. 4 and 5) were revised to state exclusion for use of a nicotine-containing product in the 7 days prior to or during participation, or current misuse of alcohol or recreational substances other than cannabis (assessed by self-report and urine toxicology results). Since polypharmacy treatment was allowed for those with recent-onset psychosis after the pilot, a seventh exclusion point was added to exclude for benzodiazepine medication use that could not be discontinued for 14 days or 5 half-lives prior to participation (due to possible influence on neuropsychological performance).

### Clinical and Cognitive Assessments

Race and ethnicity (options of African American or Black, Asian, Hispanic or Latinx, White, and other [a category in which the participant could have written in the self-identified race or ethnicity]) were collected by self-report to describe the population. There were no participants who self-identified as Hispanic or Latinx or other. Details about the onset and course of the psychotic condition, past and/or current medication use, and history of nicotine use were collected during the clinical interview and review of medical records. Nicotine use was coded into 1 of 4 categories defined by the investigative team: (1) never use, (2) last use more than 1 year ago, (3) no use in the 3 months prior to participation, and (4) no use in the week prior to participation. Daily oral antipsychotic dose was converted to the chlorpromazine (CPZ) equivalent dose using published conversions (eTable 1 in Supplement 1). Symptom burden was assessed using the Scale for the Assessment of Positive Symptoms (SAPS)^18^ and the Scale for the Assessment of Negative Symptoms (SANS).^19^ The nonglobal SAPS or SANS item scores, the composite symptom scores, and subscale scores for each of the positive symptom and negative symptoms dimensions were calculated for each patient.

Participants completed a cognitive battery (eTable 2 in Supplement 1) that remained unchanged after the published pilot phase of the study,^16^ which assessed 6 domains: processing speed, attention and working memory, verbal learning and memory, visuospatial memory, ideational fluency, and executive function. One patient with AP and 3 healthy controls did not complete the cognitive testing in the original pilot study, and 1 participant with AP had a wrist injury that precluded computation of performance in processing speed.^16^ Composite scores were generated as a mean (SE) of standardized scores from the tests within each domain (eAppendix 1 in Supplement 1). A global cognition composite score for each participant was generated as the mean of the 6 domain-specific standardized composite scores.

### Imaging

Each participant completed 1 brain MRI (eAppendix 2 in Supplement 1) and 1 [^18^F]ASEM PET scan with arterial blood sampling for plasma time-activity curve and radiolabeled metabolite measurements (eAppendix 3 in Supplement 1). We used the methods and PET scanner identical to those previously described.^16^

### Data Analysis

#### PET Kinetic Analysis

PMOD Quantification Software, version 3.7 (PMOD Technologies LLC), was used to generate the metabolite-corrected arterial input function and perform kinetic analyses. The PET data were rigidly transformed into MRI space. The binding outcome, total distribution volume (V_T_), was derived by applying Logan graphical analysis (t* = 45 minutes) to each regional time-activity curve (with the metabolite-corrected arterial input function. Total distribution volume values were derived for the primary region of interest (ROI), the hippocampus, as well as 8 secondary ROIs: thalamus, striatum, and cerebellar, temporal, occipital, cingulate, frontal, and parietal cortices (eAppendix 1 in Supplement 1). The [^18^F]ASEM V_T_ values were derived from PET images after partial volume correction (PVC).^20^ Regional [^18^F]ASEM V_T_ values derived from images without PVC were secondary outcomes.

### Statistical Analysis

Statistical analyses were completed using SAS, version 9.4 (SAS Institute Inc). Prior to statistical analyses, the assumptions for each statistical approach were examined. Assumptions of each of the analyses of covariance (ANCOVA; eg, normality), linear mixed model with repeated measures (eg, linearity, normality of residuals), and partial or partial rank correlations (eg, normality, linearity) were met. Significance was set at 2-sided *P* < .05.

To test for group differences in the hippocampal availability of the α7-nAChR, we conducted an ANCOVA where group (individuals with recent-onset psychosis and healthy controls) was the between-participants factor. Age was included as a covariate due to previously published finding of a positive correlation between age and [^18^F]ASEM V_T_.^14^ A subsequent ANCOVA was conducted to determine whether lower hippocampal availability of the α7-nAChR in individuals with recent-onset psychosis relative to healthy controls was associated with NP, AP, or both. The between-participants factor, group, for this analysis had 3 levels: NP, AP, and healthy controls. Age remained a covariate in the model. To examine group differences in [^18^F]ASEM V_T_ across all 9 brain regions (hippocampus and secondary ROIs), we used a linear mixed model with repeated measures. Primary variables of interest in the model included group (individuals with recent-onset psychosis vs healthy controls or individuals with AP or NP and healthy controls), an index variable for ROI, and the 2-way interaction. Age was a covariate in the model.

Within the individuals with recent-onset psychosis, the associations between regional [^18^F]ASEM V_T_ and each clinical measure (SAPS or SANS composite score, global cognitive composite score) were assessed using partial rank or partial correlations, with age as the covariate. The association between the presence of active antipsychotic medication use and hippocampal [^18^F]ASEM V_T_ was evaluated and then included in secondary analyses.

## Results

### Study Population

Thirty-five individuals with recent-onset psychosis (mean [SD] age, 25.3 [5.6] years) and 24 healthy controls (mean [SD] age, 25.9 [4.5] years) completed the study. Of the 59 participants (mean [SD] age, 25.5 [5.2] years), 30 (51%) were women and 29 (49%) were men. In terms of race, 27 individuals (46%) were African American or Black; 4 (7%), Asian; and 28 (47%), White. Groups were similar in age, biological sex, and race (Table 1). Most participants had never used nicotine and did not use cannabis in the month prior to participation (Table 1 and eTable 3 in Supplement 1). Among the patients, mean (SD) time since onset of psychosis was 3.2 (2.3) years; 17 had NP and 18 had AP, with 5 in the NP group and 8 in the AP group reporting no use of any psychotropic medication at time of participation (13 of 35 patients [37%]) (eTable 3 in Supplement 1). Of the 22 patients using antipsychotic medication, a parenteral formulation was used by 4 patients with NP, and the binary metric of active antipsychotic medication use was similar across the 2 patient groups (eTable 3 in Supplement 1).

**Table 1.** Clinical Characteristics and PET Parameters Among Healthy Controls and Patients With Recent-Onset Psychosis

Variable	Participant group, No. %		*P* value^a^
	Healthy control (n = 24)	With recent-onset psychosis (n = 35)	
Age, mean (SD), y	25.9 (4.5)	25.3 (5.6)	.63
Sex			
Female	13 (54)	17 (49)	.67
Male	11 (46)	18 (51)	
Race^b^			
African American or Black	7 (29)	20 (57)	.10
Asian	2 (8)	2 (6)	
White	15 (63)	13 (37)	
Nicotine use			
Never use	22 (92)	29 (83)	.39
No use in past 12 mo	0	3 (9)	
No use in past 3 mo	2 (8)	2 (6)	
No use in past week	0	1 (3)	
Urine toxicology results positive for cannabis	0	3 (9)	.17
Duration of psychosis, mean (SD), y	NA	3.2 (2.3)	NA
Current antipsychotic medication use	NA	22 (63)	NA
SAPS and SANS score, median (IQR)^c^^,^^d^			
SAPS composite	NA	0 (0-6)	NA
SANS composite	NA	19 (2-41)	NA
Global cognition composite score, mean (SD)^c^^,^^e^	111.5 (7.1)	100.8 (11.2)	<.001
Domain-specific cognitive composite scores, mean (SD)^c^^,^^f^			
Processing speed^g^	119.4 (12.4)	105.2 (14.4)	<.001
Attention and working memory	108.9 (10.0)	94.9 (14.3)	<.001
Verbal memory	108.6 (11.6)	102.1 (15.1)	.10
Visuospatial memory	117.1 (11.1)	104.0 (12.8)	<.001
Ideational fluency	110.9 (12.5)	101.4 (12.2)	.007
Executive function	103.7 (6.0)	97.5 (12.0)	.03

Abbreviations: NA, not applicable; PET, positron emission tomography; SANS, Scale for the Assessment of Negative Symptoms; SAPS, Scale for the Assessment of Positive Symptoms.

^a^ Calculated using 1-way analysis of variance, χ^2^ test, or Fisher exact test as appropriate.

^b^ There were no participants who self-identified as Hispanic or Latinx or other; therefore, only race was recorded.

^c^ Sample sizes for neuropsychological testing include 21 healthy controls and 34 patients with recent-onset psychosis.

^d^ Scores of SAPS composite range from 0 to 69, with higher scores indicating higher burden of positive symptoms of psychosis. Scores of SANS composite range from 0 to 66, with higher scores indicating higher burden of negative symptoms of psychosis.

^e^ Scores range from 95.4 to 122.6 within controls and from 75.1 to 117.8 within individuals with recent-onset psychosis, with higher scores indicating better performance.

^f^ Standardized scores were averaged across tests to compute domain-specific cognitive composite scores. Within controls, scores range from 88.3 to 140.0 in processing speed, 92.5 to 130.0 in attention and working memory, 82.5 to 125.0 in verbal memory, 85.0 to 130.0 in visuospatial memory, 83.3 to 133.3 in ideational fluency, 87.5 to 110.0 in executive function. Within patients with recent-onset psychosis, scores range from 71.7 to 128.3 in processing speed, 65.0 to 120.0 in attention and working memory, 67.5 to 127.5 in verbal memory, 77.5 to 125.0 in visuospatial memory, 78.3 to 121.7 in ideational fluency, 67.5 to 110.0 in executive function. Higher scores indicate better cognitive performance.

^g^ One patient with recent-onset psychosis had a wrist injury that precluded computation of processing speed.

The SAPS composite scores were low in participants with recent-onset psychosis (Table 1). While there was no difference in SAPS composite scores between AP and NP groups, the SANS composite score (Table 1) was higher in the NP compared with the AP groups (eTable 3 in Supplement 1). Individuals with recent-onset psychosis had lower global cognition composite scores than controls (Table 1), with lowest performance in those with NP (eTable 3 in Supplement 1).

### MRI and [^18^F]ASEM PET Imaging

**Figure 1.**
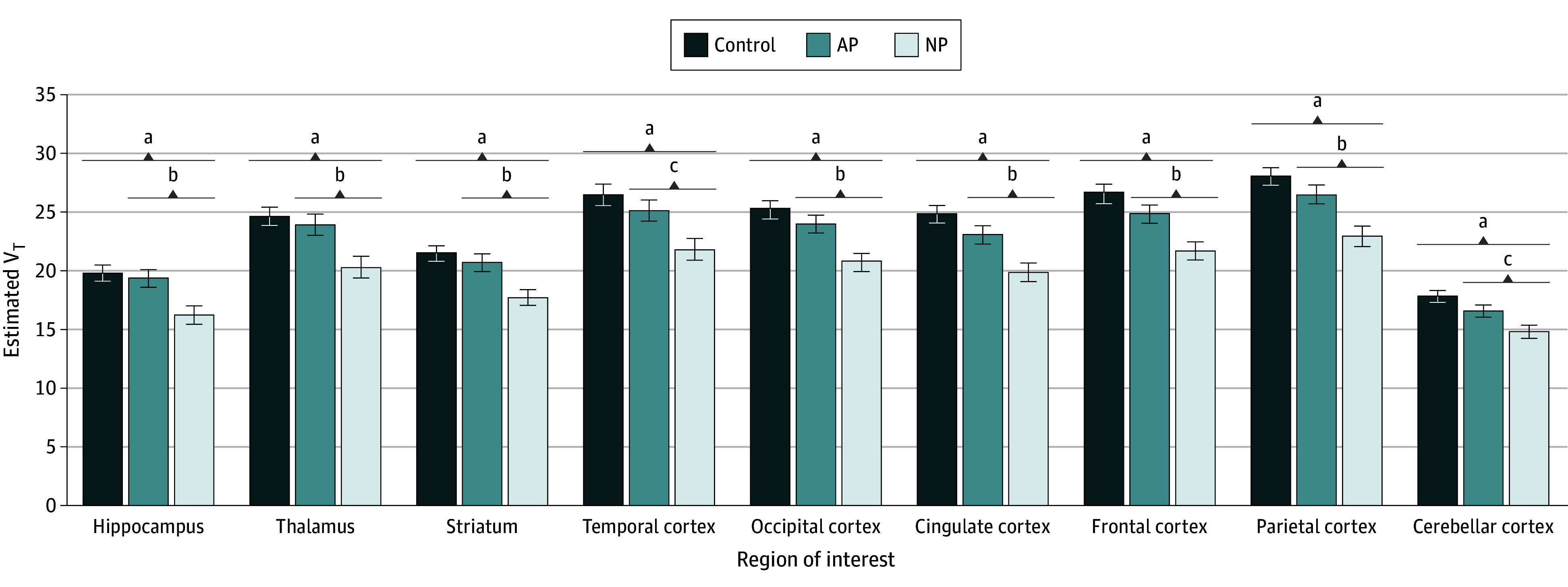
Group Comparisons Between Fluorine 18–Labeled ASEM ([^18^F]ASEM) Total Distribution Volume (V_T_) Values Estimated mean (SE) of [^18^F]ASEM V_T_ values in hippocampus (primary end point) and additional regions (secondary end points) from healthy controls (n = 24), patients with affective psychosis (AP) (n = 18), and patients with nonaffective psychosis (NP) (n = 17). V_T_ was estimated from images corrected for partial volume. ^a^*P* < .001. ^b^*P* < .01. ^c^*P* < .05.

The ROI volumes did not differ between the healthy controls and patients with recent-onset psychosis (eTable 4 in Supplement 1) or among the 3 groups (controls and those with AP or NP) (eTable 5 in Supplement 1), after adjusting for total intracranial volume and age. Descriptive mean [^18^F]ASEM V_T_ values across the hippocampus and secondary regions of interest for each group are presented in eTable 6 in Supplement 1. In age-adjusted analyses, patients with recent-onset psychosis had lower hippocampal [^18^F]ASEM V_T_ (mean [SE], 17.87 [0.60]) than healthy controls (mean [SE], 19.82 [0.73]) (*P* = .04). Differences in [^18^F]ASEM V_T_ were found among the 3 groups (NP, AP, and controls); [^18^F]ASEM V_T_ was lower in the NP group (mean [SE], 16.30 [0.83]) compared with healthy controls (*P* = .006) or compared with the AP group (mean [SE], 19.34 [0.80]) (*P* = .03). However, [^18^F]ASEM V_T_ in the AP group did not differ from that in the healthy control group. Using a linear mixed model with repeated measures, the pattern of group differences on hippocampal V_T_ was similar to those of most, but not all, secondary ROIs (F_22,54_ = 2.88; *P* < .001) (Figure 1). Estimates of [^18^F]ASEM V_T_ from images without PVC did not change these results, and the pattern of results was also unchanged when analyses were repeated without including age as a covariate or when data were limited to those from only participants without a history of nicotine use. While any antipsychotic medication use was associated with lower V_T_ across all ROIs (mean [SE], 19.0 [0.81] vs 23.5 [0.56]; *P* < .001), the inclusion of this factor did not change the pattern of results between the NP and AP groups. Among patients taking oral antipsychotic medication where the CPZ equivalent dose could be computed (n = 18), [^18^F]ASEM V_T_ in hippocampus did not correlate with CPZ equivalent dose after adjusting for age (*r* = −0.28; *P* = .28). Similarly, the CPZ equivalent dose was not associated with [^18^F]ASEM V_T_ in any of the other secondary regions.

**Figure 2.**
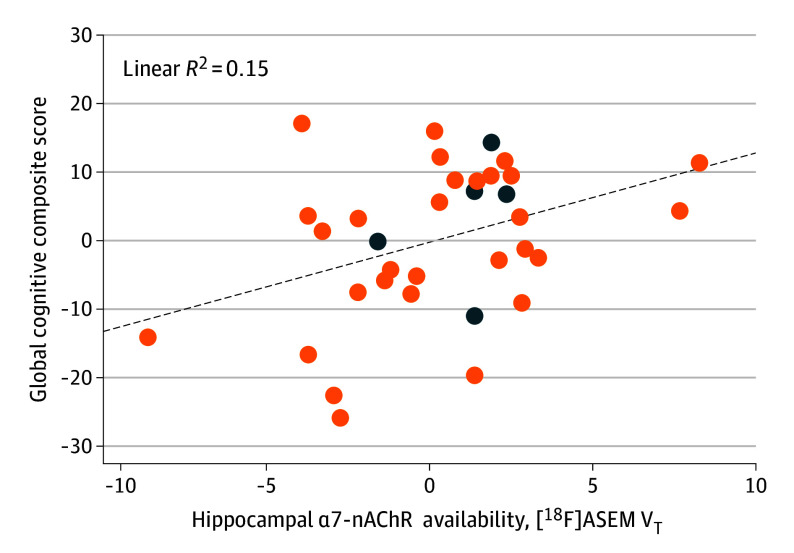
Partial Regression Plot of the Association Between Hippocampal Fluorine 18–Labeled ASEM ([^18^F]ASEM) Total Distribution Volume (V_T_) and Global Cognition Composite Score of Patients With Recent-Onset Psychosis After Adjusting for Age Data from individuals with history of nicotine use are marked (dark blue dots). Within the total study population with recent-onset psychosis (n = 35), 1 individual who also had history of nicotine use did not complete neuropsychological testing.

Among patients with recent-onset psychosis, lower hippocampal [^18^F]ASEM V_T_ was associated with a higher SAPS (*r* = −0.44; *P* = .009) but not SANS (*r* = −0.22; *P* = .20) composite score after adjusting for age (Table 2). Hippocampal [^18^F]ASEM V_T_ positively correlated with global cognition composite score after controlling for age (*r* = 0.38; *P* = .03) (Table 2 and Figure 2). The pattern of associations between [^18^F]ASEM V_T_ and clinical measures was similar across the ROIs (Table 2).

**Table 2.** Correlation of Fluorine 18–Labeled ASEM V_T_ in Hippocampus and Secondary ROIs With Clinical Symptoms and Global Cognition After Adjusting for Age

Clinical outcomes	Correlation by ROI, *r*^a^								
	Hippocampus	Thalamus	Striatum	Temporal cortex	Occipital cortex	Cingulate cortex	Frontal cortex	Parietal cortex	Cerebellar cortex
SAPS composite score	−0.44^b^	−0.45^b^	−0.45^b^	−0.36^c^	−0.42^c^	−0.48^b^	−0.48^b^	−0.46^b^	−0.39^c^
SANS composite score	−0.22	−0.23	−0.23	−0.21	−0.22	−0.18	−0.27	−0.22	−0.28
Global cognition composite score	0.38^c^	0.33	0.37^c^	0.37^c^	0.39^c^	0.37^c^	0.31	0.34	0.34

Abbreviations: ROI, region of interest; SANS, Scale for the Assessment of Negative Symptoms; SAPS, Scale for the Assessment of Positive Symptoms; V_T_, total distribution volume.

^a^ Partial rank correlation coefficients are reported for clinical symptoms and partial correlations are reported for the global cognition composite score. Hippocampal [^18^F]ASEM V_T_ values were estimated using data from images that were corrected for partial volume effects.

^b^ *P* < .01.

^c^ *P* < .05.

## Discussion

In this expanded, cross-sectional PET study, a lower hippocampal availability of the α7-nAChR was found in patients with recent-onset psychosis compared with healthy controls, largely driven by those with NP. Hippocampal α7-nAChR availability negatively correlated with the SAPS composite score, with a more specific association found between lower hippocampal α7-nAChR availability and higher hallucination subscale score. Within patients, attention and working memory performance correlated with hippocampal α7-nAChR availability, with a similar association observed across the secondary ROIs, after controlling for age. These findings generally align with a proposed working model of low α7-nAChR availability in the hippocampus of patients with psychotic conditions, wherein characteristic signs of the conditions are caused by low activity of the α7-nAChR on GABA-ergic interneurons that disinhibit excitatory inputs to glutamatergic pyramidal cells.^10^ We note that while we focused on hippocampus, results from secondary ROIs and parametric images of [^18^F]ASEM V_T_ support a more diffuse, low availability of the α7-nAChR in recent-onset psychosis, particularly in NP.

If the low α7-nAChR availability in recent-onset psychosis is validated further, it has implications for the fundamental pathophysiological understanding of nicotinic signaling in psychosis and for therapeutic strategies. Therapies in schizophrenia that target the α7-nAChR have yet to demonstrate consistent, clear efficacy.^21^ High-affinity α7-nAChR agonists or partial agonists used in phase 2 or 3 trials have failed to replicate any improvement in cognitive or negative symptoms found in the smaller, earlier studies.^22,23,24^ Similarly, AVL-3288 or JNJ-39393406, α7-nAChR type 1 positive allosteric modulators, have not yet shown cognitive benefit in patient cohorts assessed to date.^25,26^ It is possible that in NP, the low availability of this fast-desensitizing receptor limits the efficacy of α7-nAChR–targeted medications.^21^ Further mechanistic study of the role of the α7-nAChR in recent onset of psychosis and its association with cognitive or psychotic symptoms is needed to inform novel therapeutic approaches to augment α7-nAChR signaling. In addition, [^18^F]ASEM PET may prove useful to future clinical trials by providing means to characterize or enrich the trial population in terms of optimal cerebral α7-nAChR availability.

### Limitations

Study limitations included the need to expand the definition of recent-onset psychosis from 5 years (in original pilot sample) to within 10 years of psychosis onset to capture an adequately powered study sample. Despite our less stringent definition of recent-onset psychosis, our final sample included individuals with a mean (SD) of 3.2 (2.3) years since onset of psychosis. Further, 37% of patients used no antipsychotic medication at time of participation, which allowed us to examine antipsychotic medication use as a covariate. Antipsychotic use did not change the pattern of results between NP and AP. We also note that further study of the association between regional α7-nAChR availability and hallucinations would be strengthened by focus on a larger sample of patients with active positive symptom burden.

## Conclusions

The findings in this study suggest a lower hippocampal α7-nAChR availability in recent-onset psychosis, particularly among those with NP. Further study of the association between low availability of the α7-nAChR and recent-onset psychosis is warranted toward informing diagnostic or therapeutic strategies related to these findings.

## Notes

Supplement 1. **eAppendix 1.** Derivation of 6 Cognitive Domain Composite Scores and the Global Cognition Composite Score **eAppendix 2.** MRI Acquisition and Regions of Interest **eAppendix 3.** [^18^F]ASEM PET With Metabolite Measures **eTable 1.** Chlorpromazine Equivalent Doses of Oral Antipsychotic Medications **eTable 2.** Cognitive Assessments **eTable 3.** Clinical Characteristics and PET Parameters Among Healthy Controls, Patients With Recent-Onset Affective Psychosis (AP), and Patients With Recent-Onset Nonaffective Psychosis (NP) **eTable 4.** Comparison of Regional Brain Volumes Adjusted for Age and Intracranial Volume Between Healthy Controls and Individuals With Recent-Onset Psychosis **eTable 5.** Comparison of Regional Brain Volumes Adjusted for Age and Intracranial Volume Among Healthy Controls, Patients With Recent-Onset Affective Psychosis (AP), and Patients With Recent-Onset Nonaffective Psychosis (NP) **eTable 6.** Unadjusted Mean [^18^F]ASEM Total Distribution Volume (V_T_) Values for Each Region of Interest From Patients With Recent-Onset Psychosis and Healthy Controls
eReferences.

Supplement 2.
Data Sharing Statement
