# Supplementary material for: Hippocampal Availability of the α7 Nicotinic Acetylcholine Receptor in Recent-Onset Psychosis
Source: JAMA Netw Open. 2024 Aug 12;7(8):e2427163. doi: 10.1001/jamanetworkopen.2024.27163 (PMC11320165; doi:10.1001/jamanetworkopen.2024.27163)
Supplement: Supplement 1. — eAppendix 1. Derivation of 6 Cognitive Domain Composite Scores and the Global Cognition Composite Score eAppendix 2. MRI Acquisition and Regions of Interest eAppendix 3. [18F]ASEM PET With Metabolite Measures eTable 1. Chlorpromazine Equivalent Doses of Oral Antipsychotic Medications eTable 2. Cognitive Assessments eTable 3. Clinical Characteristics and PET Parameters Among Healthy Controls, Patients With Recent-Onset Affective Psychosis (AP), and Patients With Recent-Onset Nonaffective Psychosis (NP) eTable 4. Comparison of Regional Brain Volumes Adjusted for Age and Intracranial Volume Between Healthy Controls and Individuals With Recent-Onset Psychosis eTable 5. Comparison of Regional Brain Volumes Adjusted for Age and Intracranial Volume Among Healthy Controls, Patients With Recent-Onset Affective Psychosis (AP), and Patients With Recent-Onset Nonaffective Psychosis (NP) eTable 6. Unadjusted Mean [18F]ASEM Total Distribution Volume (VT) Values for Each Region of Interest From Patients With Recent-Onset Psychosis and Healthy Controls eReferences. [file jamanetwopen-e2427163-s001.pdf]

Supplementary Online Content

Wong NR, Rubin LH, Harrington CK, et al. Hippocampal availability of the α7 nicotinic acetylcholine receptor in recent-onset psychosis. *JAMA Netw Open*. 2024;7(8):e2427163. doi:10.1001/jamanetworkopen.2024.27163

eAppendix 1. Derivation of 6 Cognitive Domain Composite Scores and the Global Cognition Composite Score

eAppendix 2. MRI Acquisition and Regions of Interest

eAppendix 3. [^{18}F]ASEM PET With Metabolite Measures

eTable 1. Chlorpromazine Equivalent Doses of Oral Antipsychotic Medications

eTable 2. Cognitive Assessments

eTable 3. Clinical Characteristics and PET Parameters Among Healthy Controls, Patients With Recent-Onset Affective Psychosis (AP), and Patients With Recent-Onset Nonaffective Psychosis (NP)

eTable 4. Comparison of Regional Brain Volumes Adjusted for Age and Intracranial Volume Between Healthy Controls and Individuals With Recent-Onset Psychosis

eTable 5. Comparison of Regional Brain Volumes Adjusted for Age and Intracranial Volume Among Healthy Controls, Patients With Recent-Onset Affective Psychosis (AP), and Patients With Recent-Onset Nonaffective Psychosis (NP)

eTable 6. Unadjusted mean [^{18}F]ASEM Total Distribution Volume (V_T) Values for Each Region of Interest From Patients With Recent-Onset Psychosis and Healthy Controls

eReferences.

This supplementary material has been provided by the authors to give readers additional information about their work.

© 2024 Wong NR et al. *JAMA Network Open*.

eAppendix 1. Derivation of 6 Cognitive Domain Composite Scores and the Global Cognition Composite Score

Raw test scores derived across the nine cognitive tests (eTable 2) were transformed into scaled scores (M = 10 and SD = 3) according to the frequencies of different raw scores in the underlying normative sample [Calibrated Neuropsychological Normative System™ (PAR Inc, Lutz, FL)]. Scaled scores within each cognitive domain were then averaged to compute the six domain-specific composite scores and expressed as domain-specific standardized composite scores (M=100, SD=15). The domain-specific standardized composite scores were averaged to compute the global cognition composite score.

eAppendix 2. MRI Acquisition and Regions of Interest

Each participant completed a Structural T1-weighted Magnetization-Prepared Rapid Gradient-Echo (MPRAGE) sequence of the brain at 3 Tesla (0.75 x 0.75 x 0.8 mm voxel size). Depending on scanner availability, the MPRAGE was acquired on either a Philips Ingenia CX dStream or a Phillips Elition scanner (Philips Healthcare, Amsterdam, Netherlands). Brain regions were delineated from MPRAGE data using the FreeSurfer image analysis suite, version 5. Freesurfer was also used to segment the total intracranial volume. ROI volumes adjusted for total intracranial volume and age were compared between groups.

eAppendix 3. [^{18}F]ASEM PET With Metabolite Measures

[^{18}F]ASEM was synthesized[1] in the Johns Hopkins PET Center and met U.S. Pharmacopeia Convention Chapter <823> acceptance testing criteria. [^{18}F]ASEM was delivered by slow intravenous push at the beginning of emission data collection. A brain-dedicated High Resolution Research Tomograph (Siemens Healthcare, Knoxville, TN) was used. Each

© 2024 Wong NR et al. *JAMA Network Open*.

participant was fitted with a thermoplastic facemask that was worn for head fixation throughout the transmission and emission scanning. The mean molar activity of [^{18}F]ASEM was 2414.1 ± 1945.8 GBq per micromole (65,247.1 ± 52,588.1 mCi per micromole) at time of injection. The mean injected dose was 525.9 ± 19.8 MBq. The 90 min list mode data were binned into 30 frames and reconstructed using the iterative ordered subsets expectation maximization algorithm.[2] The relative percent of [^{18}F]ASEM and its radiometabolites in blood plasma was measured at 5, 10, 20, 30, 60, 75 and 90 min after injection using reverse phase-high performance liquid chromatography[3] as previously described.[4] Radiometabolite-corrected plasma time-activity curves (TACs) were then derived using PMOD (v3.7, PMOD Technologies Ltd, Zurich, Switzerland) by applying the relative percent of parent [^{18}F]ASEM time-profiles to the total plasma TACs after linear interpolation.

© 2024 Wong NR et al. *JAMA Network Open*.

eTable 1. Chlorpromazine Equivalent Doses of Oral Antipsychotic Medications

**Antipsychotic medication**	**CPZ equivalent dose**
Aripiprazole	7.5
Brexipiprazole	1
Cariprazine	1.5
Loxapine	10
Olanzapine	5
Quetiapine	75
Risperidone	2

Adult oral daily dose equivalents expressed as the dose equivalent to chlorpromazine 100mg daily.[5-7]

© 2024 Wong NR et al. *JAMA Network Open*.

eTable 2. Cognitive Assessments

Cognitive Domain	Test	Test Index Used
Processing Speed	GPB[8]	Mean completion time over two trials bilaterally
	PCT[9]	Sum of letter completions
		Sum of pattern completions
Attention and Working Memory	DS[10]	Sum of longest forward and backward span
	BTA[11]	Sum of total letters and numbers correct
Auditory-verbal Learning/Memory	HVLT[12]	Total words learned (HVLT Learning)
		Delayed word recall (HVLT delay)
Visuospatial Learning/Memory	BVMT[13]	Total figures learned (BVMT Learning)
		Delayed figure recall (BVMT delay)
Ideational Fluency	CIFA-VF[14]	Letter-guided verbal fluency (S and P)
		Category-guided verbal fluency (Animals and Supermarkets)
	CIFA-DF[14]	Novel designs produced
Executive Functioning	M-WCST[15]	Number of category sorts
		Number of perseverative errors

Abbreviations: BTA, Brief Test of Attention; BVMT, Brief Visuospatial Memory Test – Revised; CIFA, Calibrated Ideational Fluency Assessment (VF, Verbal Fluency; DF, Design Fluency (DF) subtests of the CIFA); DS, Digit Span Forward and Backward; GPB, Grooved Pegboard Test; HVLT, Hopkins Verbal Learning Test – Revised; MWCST, Modified Wisconsin Card Sorting Test (MWCST; Nelson, 1976); PCT, Salthouse Perceptual Comparison Test

© 2024 Wong NR et al. *JAMA Network Open*.

eTable 3. Clinical Characteristics and PET Parameters Among Healthy Controls, Patients With Recent-Onset Affective Psychosis (AP), and Patients With Recent-Onset Nonaffective Psychosis (NP)

	Control (N = 24) N (%)	AP (N = 18) N (%)	NP (N=17) N (%)	*P*[a]
**Age (years), M (SD)**	25.9 (4.5)	26.4 (5.5)	24.1 (5.7)	0.37
**Sex**				0.08
**Female**	13 (54)	12 (67)	5 (29)	
**Male**	11 (46)	6 (33)[f]	12 (71)	
**Race**				0.09
**African American/Black**	7 (29)	9 (50)	11 (65)	
**Asian**	2 (8)	0 (0)	2 (12)	
**White**	15 (63)	9 (50)	4 (23)	
**Nicotine use**				0.08
**Never**	22 (92)	15 (83)	14 (82)	
**No use in past 12 month**	0 (0)	3 (17)	0 (0)	
**No use in past 3 month**	2 (8)	0 (0)	2 (12)	
**No use in past week**	0 (0)	0 (0)	1 (6)	
**Urine toxicology positivity for cannabis**	0 (0)	1 (6)	2 (12)	0.28
**Years of Psychosis, M (SD)**	-	3.2 (1.8)	3.3 (2.8)	0.82
**Current antipsychotic medication use**	-	10 (56)	12 (71)	0.36
**SAPS/SANS, median (IQR)**				
**SAPS composite**	-	0 (0)	4 (13)	0.17
**SANS composite**	-	4.5 (19)	39.0 (30.5)	<0.001
**Global Cognition Composite Score, M (SD)**[b]	111.5 (7.1)	106.5 (7.5)	95.1 (11.6)	<0.001
**Domain-specific cognitive composite scores, M (SD)**[b]				
**Processing Speed**	119.4 (12.4)[d]	110.5 (10.3)	100.2 (16.1)[c,e]	<0.001
**Attention/Working Memory**	108.9 (10.0)[d]	102.1 (11.6)	87.8 (13.3)[c,e]	<0.001
**Verbal Memory**	108.6 (11.6)	109.1 (8.8)	95.1 (17.1)[c,e]	0.003
**Visuospatial Memory**	117.1 (11.1)[d]	107.8 (9.3)	100.3 (14.9)[c]	<0.001
**Ideational Fluency**	110.9 (12.5)[d]	107.2 (8.8)	95.6 (12.6)[c,e]	<0.001
**Executive Function**	103.7 (6.0)[d]	103.2 (9.1)	91.8 (12.0)[c,e]	<0.001

[a]*P* values for one way ANOVA, Chi-Square, or Fisher's Exact Test as appropriate. [b]One patient and three healthy controls did not complete neuropsychological testing in the pilot study, resulting in the following sample sizes with neuropsychological testing data: healthy controls (N=21), AP (N=17), NP (N=17). One participant with AP had a wrist injury that precluded computation of performance in processing speed. Standardized scores were averaged across tests to compute domain-specific factor scores. [c]*P* <0.05 versus controls; [d]*P* <0.05 versus all patients

© 2024 Wong NR et al. *JAMA Network Open*.

(AP + NP) [e]*P* <0.05 versus AP. Abbreviations: IQR=interquartile range; M=mean; SD=standard deviation

© 2024 Wong NR et al. *JAMA Network Open*.

eTable 4. Comparison of Regional Brain Volumes Adjusted for Age and Intracranial Volume Between Healthy Controls and Individuals With Recent-Onset Psychosis

Region of Interest	Control (N = 24) M (SE)	Recent-onset Psychosis (N = 35) M (SE)	β (95% CI)	*P*[a]
Hippocampus	8.32 (0.19)	8.05 (0.16)	-0.27 (-0.77, 0.22)	0.28
Thalamus	16.10 (0.28)	15.73 (0.23)	-0.40 (-1.11, 0.37)	0.32
Striatum	19.67 (0.36)	19.73 (0.30)	0.06 (-0.88, 1.01)	0.89
Temporal Cortex	95.20 (3.03)	88.55 (2.51)	-6.64 (-14.55, 1.26)	0.09
Occipital Cortex	43.30 (0.99)	44.25 (0.83)	-1.05 (-3.65, 1.55)	0.42
Cingulate Cortex	20.22 (0.43)	20.15 (0.36)	-0.06 (-1.18, 1.06)	0.56
Frontal Cortex	173.38 (3.17)	173.41 (2.63)	0.03 (-8.23, 8.29)	0.99
Parietal Cortex	111.14 (2.38)	109.19 (1.97)	-1.94 (-8.14, 4.25)	0.53
Cerebellar Cortex	102.91 (2.99)	96.93 (2.48)	-5.98 (-13.77, 1.82)	0.13

β=unstandardized beta coefficient which reflects the mean difference between recent-onset psychosis in each region of interest. CI=confidence interval. [a]*P* values for group differences from a single linear mixed model with repeated measures. Adjusted regional volumes are listed as M=mean (SE=standard error) and presented in cm^3.

© 2024 Wong NR et al. *JAMA Network Open*.

eTable 5. Comparison of Regional Brain Volumes Adjusted for Age and Intracranial Volume Among Healthy Controls, Patients With Recent-Onset Affective Psychosis (AP), and Patients With Recent-Onset Nonaffective Psychosis (NP)

Region of Interest	Control (N = 24) M (SE)	AP (N = 18) M (SE)	NP (N=17) M (SE)	AP (vs. Control) β (95% CI)	NP (vs. control) β (95% CI)	AP (vs. NP) β (95% CI)
Hippocampus	8.32 (0.19)	8.19 (0.22)	7.90 (0.23)	-0.13 (-0.71, 0.45)	-0.42 (-1.01, 0.17)	0.29 (-0.34, 0.92)
Thalamus	16.10 (0.28)	15.87 (0.37)	15.58 (0.38)	-0.23 (-1.24, 0.75)	-0.52 (-1.51, 0.47)	0.29 (-0.76, 1.35)
Striatum	19.67 (0.36)	19.95 (0.44)	19.49 (0.45)	0.29 (-0.87, 1.45)	-0.17 (-1.35, 1.01)	0.46 (-0.80, 1.71)
Temporal Cortex	95.20 (3.03)	89.66 (3.77)	87.39 (3.88)	-5.54 (-15.56, 4.75)	-7.81 (-17.99, 2.38)	2.26 (-8.60, 13.13)
Occipital Cortex	43.30 (0.99)	44.24 (1.37)	44.25 (1.41)	-1.06 (-4.69, 2.57)	-1.05 (-4.74, 2.64)	-0.01 (-3.94, 3.93)
Cingulate Cortex	20.22 (0.43)	19.80 (0.60)	20.54 (0.62)	-0.42 (-2.02, 1.18)	0.32 (-1.32, 1.94)	-0.73 (-2.47, 1.01)
Frontal Cortex	173.38 (3.17)	172.87 (4.75)	173.98 (4.88)	-0.51 (-13.10, 12.09)	0.60 (-12.20, 13.41)	-1.11 (-14.77, 12.55)
Parietal Cortex	111.14 (2.38)	105.16 (3.15)	113.46 (3.24)	-5.98 (-14.32, 2.37)	2.33 (-6.16, 10.81)	-8.30 (-17.36, 0.75)
Cerebellar Cortex	102.91 (2.99)	98.66 (4.07)	95.10 (4.19)	-4.25 (-15.04, 6.55)	-7.80 (-18.79, 3.17)	3.56 (-8.15, 15.27)

β=unstandardized beta coefficient which reflects the mean difference between groups. CI=confidence interval. Adjusted regional volumes are listed as M=mean (SE=standard error) and presented in cm^3.

© 2024 Wong NR et al. *JAMA Network Open*.

eTable 6. Unadjusted Mean [^{18}F]ASEM Total Distribution Volume (V_T) Values for Each Region of Interest From Patients With Recent-Onset Psychosis and Healthy Controls

Region of Interest	Recent-onset Psychosis (N = 35)	Control (N = 24)	AP (N = 18)	NP (N=17)
Hippocampus	17.85 (3.65)	19.85 (3.40)	19.37 (2.76)	16.25 (3.86)
Thalamus	22.16 (4.57)	24.65 (3.34)	23.93 (3.87)	20.29 (4.60)
Striatum	19.26 (3.60)	21.54 (2.88)	20.68 (2.64)	17.75 (3.93)
Temporal Cortex	23.51 (4.33)	26.61 (3.79)	25.11 (3.74)	21.82 (4.35)
Occipital Cortex	22.40 (3.96)	25.26 (3.02)	23.97 (3.55)	20.74 (3.78)
Cingulate Cortex	21.52 (4.14)	24.89 (2.97)	23.07 (3.59)	19.88 (4.14)
Frontal Cortex	23.32 (3.85)	26.63 (3.40)	24.83 (2.89)	21.72 (4.17)
Parietal Cortex	24.76 (4.20)	28.03 (3.35)	26.48 (3.35)	22.94 (4.32)
Cerebellar Cortex	15.72 (2.63)	17.86 (1.92)	16.57 (2.45)	14.82 (2.59)

The right-most two columns display mean [^{18}F]ASEM total distribution volume (V_T) values after subdividing the recent-onset psychosis group into individuals with recent-onset of affective psychosis (AP), and patients with recent-onset of non-affective psychosis (NP). [^{18}F]ASEM V_T values were estimated using data from images that were corrected for partial volume effects. V_T values are in units of mL cm^{-3} and are presented as Mean (Standard Deviation).

© 2024 Wong NR et al. *JAMA Network Open*.

eReferences.

1. Gao Y, Kellar KJ, Yasuda RP, et al. Derivatives of dibenzothiophene for positron emission tomography imaging of α7-nicotinic acetylcholine receptors. *Journal of medicinal chemistry*. Oct 10 2013;56(19):7574-89. doi:10.1021/jm401184f

2. Rahmim A, Cheng JC, Blinder S, Camborde ML, Sossi V. Statistical dynamic image reconstruction in state-of-the-art high-resolution PET. *Physics in medicine and biology*. Oct 21 2005;50(20):4887-912. doi:10.1088/0031-9155/50/20/010

3. Hilton J, Yokoi F, Dannals RF, Ravert HT, Szabo Z, Wong DF. Column-switching HPLC for the analysis of plasma in PET imaging studies. *Nuclear medicine and biology*. Aug 2000;27(6):627-30. doi:10.1016/s0969-8051(00)00125-6

4. Coughlin JM, Du Y, Rosenthal HB, et al. The distribution of the alpha7 nicotinic acetylcholine receptor in healthy aging: An in vivo positron emission tomography study with [(18)F]ASEM. *NeuroImage*. Jan 15 2018;165:118-124. doi:10.1016/j.neuroimage.2017.10.009

5. Woods SW. Chlorpromazine equivalent doses for the newer atypical antipsychotics. *The Journal of clinical psychiatry*. Jun 2003;64(6):663-7. doi:10.4088/jcp.v64n0607

6. Ray WA, Fuchs DC, Olfson M, et al. Antipsychotic Medications and Mortality in Children and Young Adults. *JAMA psychiatry*. Nov 29 2023;doi:10.1001/jamapsychiatry.2023.4573

7. Pharmacists CoPaN. Psychiatric pharmacy essentials: antipsychotic dose equivalents. . . Accessed January 16, 2024, https://cpnp.org/guideline/essentials/antipsychotic-dose-equivalents

8. Merker B, Podell K. Grooved Pegboard Test. In: Kreutzer JS, DeLuca J, Caplan B, eds. *Encyclopedia of Clinical Neuropsychology*. Springer New York; 2011:1176-1178.

9. Salthouse TA. The processing-speed theory of adult age differences in cognition. *Psychological review*. Jul 1996;103(3):403-28. doi:10.1037/0033-295x.103.3.403

10. Wechsler D. Wechsler adult intelligence scale. *Archives of Clinical Neuropsychology*. 1955;

11. McLaren ME, Woods AJ. Brief Test of Attention. In: Kreutzer J, DeLuca J, Caplan B, eds. *Encyclopedia of Clinical Neuropsychology*. Springer International Publishing; 2017:1-2.

12. Shapiro AM, Benedict RH, Schretlen D, Brandt J. Construct and concurrent validity of the Hopkins Verbal Learning Test-revised. *The Clinical neuropsychologist*. Aug 1999;13(3):348-58. doi:10.1076/clin.13.3.348.1749

13. Benedict RHB, Schretlen D, Groninger L, Dobraski M, Shpritz B. Revision of the Brief Visuospatial Memory Test: Studies of normal performance, reliability, and validity. *Psychological Assessment*. 1996;8(2):145-153. doi:10.1037/1040-3590.8.2.145

© 2024 Wong NR et al. *JAMA Network Open*.

14. Schretlen D, Vannorsdall T. Calibrated ideational fluency assessment (CIFA) professional manual. *Psychological Assessment Resources*. 2010;

15. Nelson HE. A modified card sorting test sensitive to frontal lobe defects. *Cortex; a journal devoted to the study of the nervous system and behavior*. Dec 1976;12(4):313-24. doi:10.1016/s0010-9452(76)80035-4

© 2024 Wong NR et al. *JAMA Network Open*.
