# Supplementary material for: Hippocampal Availability of the α7 Nicotinic Acetylcholine Receptor in Recent-Onset Psychosis
Source: JAMA Netw Open. 2024 Aug 12;7(8):e2427163. doi: 10.1001/jamanetworkopen.2024.27163 (PMC11320165; doi:10.1001/jamanetworkopen.2024.27163)
Supplement: Supplement 2. — Data Sharing Statement [file jamanetwopen-e2427163-s002.pdf]

Data Sharing Statement

Wong. Hippocampal Availability of the α7 Nicotinic Acetylcholine Receptor in Recent-Onset Psychosis. *JAMA Netw Open*. Published August 12, 2024.
doi:10.1001/jamanetworkopen.2024.27163

Data
Data available: Yes
Data types: Deidentified participant data
How to access data: jcoughl2@jhmi.edu
When available: With publication

Supporting Documents
Document types: None

Additional Information
Who can access the data: researchers whose proposed use of the data has been approved
Types of analyses: for a specified purpose
Mechanisms of data availability: with a signed data access agreement
Any additional restrictions: N/A
